# In Silico Simulation of the Systemic Drug Exposure Following the Topical Application of Opioid Analgesics in Patients with Cutaneous Lesions

**DOI:** 10.3390/pharmaceutics13020284

**Published:** 2021-02-21

**Authors:** Maksim Khotimchenko, Victor Antontsev, Kaushik Chakravarty, Hypatia Hou, Jyotika Varshney

**Affiliations:** VeriSIM Life Inc., 1 Sansome St, Suite 3500, San Francisco, CA 94104, USA; maksim.khot@verisimlife.com (M.K.); victor.antontsev@verisimlife.com (V.A.); kaushik.chakravarty@verisimlife.com (K.C.); hypatia.hou@verisimlife.com (H.H.)

**Keywords:** computational modeling, opioid analgesics, transdermal, pharmacokinetics

## Abstract

The use of opioid analgesics in treating severe pain is frequently associated with putative adverse effects in humans. Topical agents that are shown to have high efficacy with a favorable safety profile in clinical settings are great alternatives for pain management of multimodal analgesia. However, the risk of side effects induced by transdermal absorption and systemic exposure is of great concern as they are challenging to predict. The present study aimed to use “BIOiSIM” an artificial intelligence-integrated biosimulation platform to predict the transdermal disposition of opioid analgesics. The model successfully predicted their exposure following the topical application of central opioid agonist buprenorphine and peripheral agonist oxycodone in healthy human subjects with simulation of intra-skin exposure in subjects with burns and pressure wounds. The predicted plasma levels of analgesics were used to evaluate the safety of the therapeutic pain control in patients with the dermal structural impairments caused by acute (burns) or chronic cutaneous lesions (pressure wounds) with topical opioid analgesics.

## 1. Introduction

**Figure 1:**
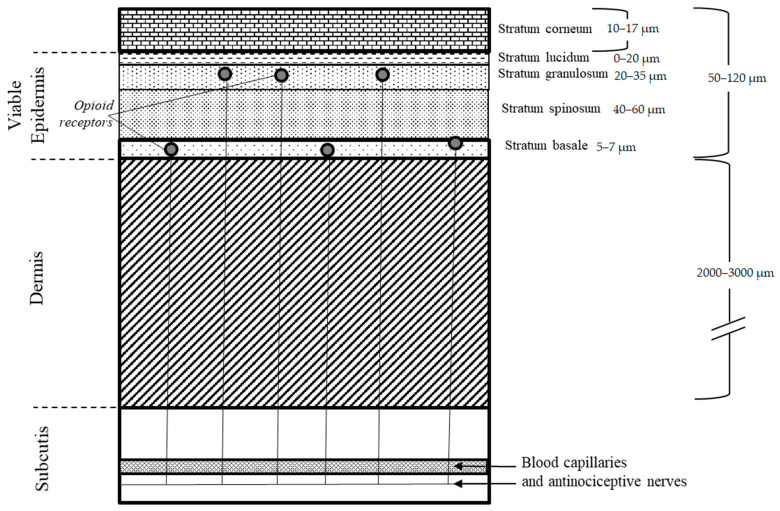
General schematic of the skin layers with peripheral opioid receptors located in the epidermis layer [3,4].

Management of moderate and severe pain poses a major challenge in the industry as it is by a partially subjective phenomenon that is associated with actual or potential damage to tissues resulting from illness or injury. This syndrome is typically observed in almost any disease as a manifestation of a patient’s worsening condition and corresponding decrease in the quality of life. Depending on the severity, treatment of pain involves the usage of opioid analgesics, nonsteroidal anti-inflammatory drugs (NSAIDs), and adjuvant analgesics [1]. Light and mild pain syndrome may be successfully alleviated either with NSAID or through the application of local anesthetics. However, management of the severe pain usually requires administration of narcotic opioid analgesics due to their focused antinociceptive mechanism of action and substantially higher potency. They interact with specific receptors that, when activated, suppress the transmission of pain impulses. Their inhibitory activity is exerted in the brain and by increasing the threshold of nociceptive fibers in the substantia gelatinosa in the spinal cord’s dorsal horns. Nevertheless, the usage of opioids is limited because of the obvious adverse effects mostly associated with their central activity and a fear of addiction among patients [2]. Numerous studies over the last two decades have shown that opioid receptors are also present in the peripheral nervous system and located in stratum granulosum and stratum basale of epidermis (Figure 1) [3,4]. This provides a scientific base for the topical use of opioid analgesics in severe pain management [5].

Among the myriads of physical or physiological insults that can stimulate nociceptors, acute thermal skin injuries and pressure ulcers are usually responsible for the most intensive pain syndrome. Due to the extreme degree of the pain associated with those skin lesions, opioids remain the mainstay of treatment, especially in the acute phase of the burn disease or long-lasting pressure wounds [6]. The topical application of the opioid analgesics may serve as a promising alternative approach for pain relief because it is supposed to be associated with a lower risk of adverse effects and sufficient potency. Noninvasive methods of drug administration typically offer avoidance of systemic exposure and undesirable effects induced activated central opioid receptors in thalamus. It should be noted that the transdermal administration is a common route for the majority of the opioids, as they easily penetrate the skin barrier and are then absorbed into the blood flow through a network of capillaries located in subcutaneous tissue. Upon absorption, they are almost immediately transported to the opioid receptors localized in the central nervous system (CNS) providing both their therapeutic activity and associated adverse effects. Passing through the skin layers, the molecules of analgesics stimulate dermal opioid receptors inducing local analgesia. This effect directly depends on the drug skin concentration [7]. Thus, the attention should be given to the skin retention properties of an analgesic drug compound. Considering local analgesia as a beneficial antinociceptive mechanism, the often reported transdermal penetration should be seen as a negative feature obviously related to the systemic drug exposure and induction adverse effects. The rational choice of an opioid for topical analgesia should involve drugs with the highest retention in epidermis and the low skin permeation capacity. Furthermore, the selection should consider drug affinity to the opioid receptors localized in the skin epidermis [8].

Clinical evaluation of drug pharmacokinetics in patients with severe skin lesions is substantially hindered due to unstable patient conditions and dramatically altered biochemical pathways [9]. Animal models, on the other hand, were shown to be irrelevant due to significant differences in the composition of dermal layers, the density of hair follicles, and skin thickness. That inevitably leads to distinct absorption profiles in animal species when compared to the human ones [10]. Among the possible solutions, computational modeling is thought to be one of the most promising. It has been previously applied to predict and simulate systemic exposure following topical application of the drug compounds [11]. Most current transdermal models are complex and consider skin as a series of interconnected compartments. Modeled parameters of the skin layers for different species, subjects, and pathological alterations significantly vary, resulting in the highly variable results [12].

A key challenge with studying this type of dosing regimen comes with the high variability in experimental methods for studying skin concentration of compounds, identification of patient cohorts for studies, and overall challenge with obtaining experimental measurements that adequately characterize local, intra-skin permeation of compounds. Computational tools can aid in this interrogation of potential phenomena by using hybrid mechanistic models that capture relevant physics augmented with artificial intelligence (AI) and quantitative system-property/activity relationship models (QSPR/QSAR) [13]. These methodologies bypass the need for experimentation, and can inform clinician and scientist strategies for optimizing dosage forms and regimens in specific patients with compromised skin integrity. There is a diversity of models that exist; however, they traditionally predict layer-specific permeability and are trained on in vitro data of decoupled phenomena, or do not incorporate methods for resolving identifiability challenges [14,15,16]. An approach that combines these methodologies with in vivo data for training stands to improve the accuracy and relevance of insight pertaining to skin permeation of topically-applied compounds. To that end, VeriSIM Life (VSL) has expanded its in silico pharmacology simulation platform, BIOiSIM. The platform provides a scalable computational approach through the integration of AI into physiological modeling to make accurate and faster predictions that can be applied to and trained on large compound datasets. The integration of machine learning (ML) with mechanistic modeling allows BIOiSIM to fill in missing data gaps commonly found in biological datasets through a combination of parameter optimization and prediction. To validate, train, and test the model, a dataset comprising distinct drug compounds with common prevalent topical application was chosen to estimate their local analgesic potential. Two opioid analgesics drugs proposed for transdermal administration in the antipain therapy, namely oxycodone and buprenorphine, were used in the present study. Buprenorphine is utilized in the form of a topical patch for treatment of moderate to severe cancer pain, and appears to induce dose-dependent relief for musculoskeletal pain [17]. Results of the clinical studies confirm that oxycodone possesses significant analgesic activity being dissolved in water and then mixed with the debridement ointment and applied to the skin lesions. This gave almost immediate and complete relief from pain [18]. In addition, there is evidence from animal studies that most opioids (apart from buprenorphine and oxycodone) induce some degree of suppression of the immune system through modification of natural killer (NK) cells function, lymphocytes T, action of interleukin–2, or interferon gamma. Lack of negative impact on the immune system may improve safety of the drug regarding infections and cancer dissemination [19]. Transdermal disposition and intra-skin concentrations were evaluated to assess the risk/benefit ratio for administering these opioids to healthy patients and to patients with thermal and pressure skin lesions. This study was aimed to use a simple and physiologically relevant transdermal model approach, developed using BIOiSIM, for prediction of the possible systemic exposure of opioid analgesics that were shown to exert local analgesia via activation of the skin opioid receptors in healthy and damaged skin.

## 2. Materials and Methods

### 2.1. BIOiSIM Model Expansion

BIOiSIM is a software platform comprised of semi-mechanistic models of in vivo physiology with 16 individual compartments corresponding to critical tissues and organs in the body. Ordinary differential equations (ODEs) are used to model the interactions between the different compartments in both the pharmacokinetic (PK) and pharmacodynamic (PD) contexts [20,21]. Model inputs include subject-specific parameters (organ volumes, blood flow rates, tissue composition, enzyme expression levels), relevant PK mechanisms (clearance, drug dissolution, permeability), among others. The core framework of the model has been discussed in previous publications [20,21]. The model used for characterizing skin permeability has previously been validated for simulating systemic disposition of transdermally-applied drugs; the work here expands on the systems biology model previously described [11]. 

When viewing the skin as a single, inactive barrier (i.e., no clearance or active transport), two specific parameters can describe the nature of time-dependent kinetics and extent of transdermal absorption of a compound: the skin barrier permeability (*k_perm_*) and compound dermal bioavailability (*F_derm_*). Changing environmental factors, such as humidity, addition of permeation enhancers, and skin composition can influence these coefficients. The relationship between these compounds and their overall absorption rate of a compound can be captured as:

(1) $$\frac{dA}{dt}=SA*Dose*F_{derm}*k_{perm}$$

where *dA/dt* represents the overall rate of mass transport, and *SA* is the surface area of formulation application.

#### 2.1.1. Intra-Skin Permeability Prediction

**Figure 2:**
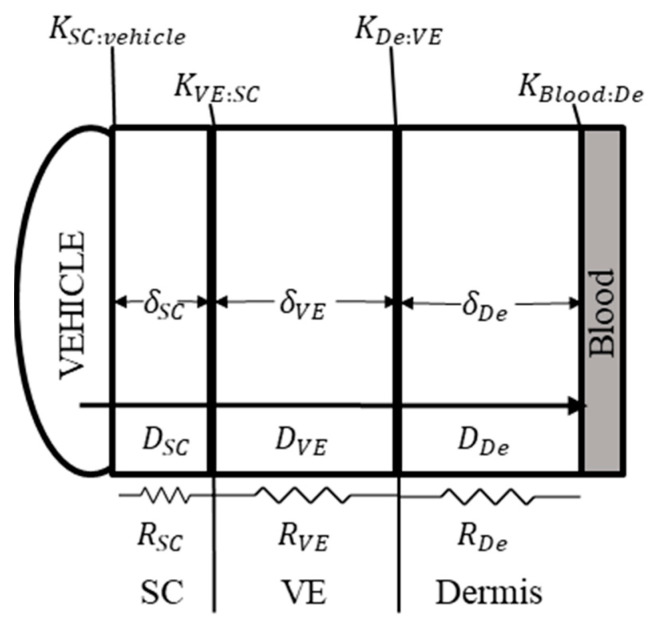
Basic diagram of 1D model for simulating transdermal permeation in BIOiSIM. D_i_ is the diffusion coefficient for a compound across each respective skin layer; K_i:g_ is the partition coefficient boundary condition at each layer interface; R_i_ is the resistance to permeation; and δ is the thickness of each layer. SC = Stratum corneum; VE = viable epidermis; De = Dermis.

Extension of the model for intra-skin prediction requires estimation of additional parameters that are not experimentally available—specifically, diffusion coefficient (diffusivity, *D*) and partition coefficient (*K*) values for layers with assumed independent behavior. The relevant systems are captured in Figure 2, and the key assumptions in the model are documented in Supplementary Table S1.

The permeability of compounds is dependent on both the thickness and diffusivity within a layer, as outlined in Fick’s first law [22,23]. Thus, the permeability of each individual layer can be calculated as:

(2) $$k_{perm,\;\;i}=\frac{D_{i}}{\delta_{i}}$$

where *i* refers to a respective skin layer, *D* is the diffusivity, and *δ* is the thickness of the layer. Key assumptions are made to simulate the skin accurately and reduce reliance on immeasurable parameter values. The layers were assumed to behave as a system of serially connected resistors, each with a permeation resistance *R*. Stratum corneum (SC) permeation was assumed to be dominated via lipid channels between the packed corneocytes for both selected compounds. The skin was split up into three layers for simulation—the stratum corneum, viable epidermis, and dermis. Thus, the relationship between layer resistance and permeability can be defined as:

(3) $$R=\frac{1}{k_{perm}}$$

(4) $$R_{total}=R_{SC}+R_{VE}+R_{De}$$

(5) $$\frac{1}{k_{perm}}=\frac{1}{k_{perm,SC}}+\frac{1}{k_{perm,\;VE}}+\frac{1}{k_{perm,\;De}}$$

where *R* is the permeation resistance for each respective layer, and *SC*, *VE*, and *De* refer to stratum corneum, viable epidermis, and dermis, respectively. Thus, with a known total skin permeability, diffusivity of individual skin layers, and their thickness, we can estimate the permeability within each skin layer with some confidence and make assessments of compound permeation to site-specific areas. The skin thicknesses utilized in the simulation were 0.3 cm (3000 micron) for the dermis, 0.01 cm (100 micron) for the viable epidermis, and 0.002 cm (20 micron) for the stratum corneum as reasonable estimates from literature (Figure 1) [3,4]. Skin was assumed to be well hydrated. Diffusion within the formulation was assumed to be fast and non-rate limiting, and dermal bioavailability was assumed to be independent of the stratum corneum.

Diffusivity for each of the individual layers was predicted using an optimized overall skin permeability, *k_perm_*, and normalization of calculated permeability values using known quantitative-structure property relationship models (QSPR) [22,24]. Given the similarity in composition between the two layers, the standard assumption of equivalent diffusivity in viable epidermis and dermis is made. Partition coefficients (Figure 2) for the four barriers are also calculated via established QSPR models [7,24]. Although there are no direct models for predicting the partition coefficient of viable epidermis to stratum corneum, this value was estimated as:

(6) $$K_{VE:SC}=\frac{K_{VE:water}}{K_{SC:water}}$$

The partition coefficient between viable epidermis and dermis was assumed to equal 1, as they are not traditionally evaluated separately for compound partitioning [25]. All physiological parameters characterizing layer density and mass fraction composition influencing partitioning were obtained from the original source [7,24,26]. The vehicle used in the formulation was assumed to be similar to water with respect to its partitioning behavior.

#### 2.1.2. Calculation of Intra-Skin Concentration

The concentration of compound at the apical surface of each layer was calculated. It was assumed that the mu-opioid receptors that are targeted by both buprenorphine and oxycodone are located between the apical side of the viable epidermis and the dermis. Following the defined boundary conditions, and assuming that diffusion is dominated unidirectionally (skin concentration of compound significantly greater than plasma), we obtain the following relationships for skin concentration in healthy patients:

(7) $$C_{SC}=C_{Dose}*K_{SC:Vehicle}*F_{derm}$$

(8) $$C_{VE}=C_{SC}*\left(1-\frac{R_{SC}}{R_{total}}\right)*K_{VE:SC}$$

(9) $$C_{De}=C_{SC}*\left(1-\frac{R_{VE}+R_{SC}}{R_{total}}\right)*K_{VE:SC}*K_{De:VE}$$

In patients with lesions, it is assumed that the stratum corneum is completely disrupted while the viable epidermis and dermis stay intact [27,28]. Dermal bioavailability is maintained as constant (with the assumption that the stratum corneum does not impact compound clearance or significant retention of the compound). Thus, the relationships above are adjusted as follows for patients with lesions at the site-of-administration:

(10) $$C_{VE}=C_{Dose}*K_{VE:Vehicle}*F_{derm}$$

(11) $$C_{De}=C_{VE}*\left(1-\frac{R_{VE}}{R_{VE}+R_{De}}\right)*K_{De:VE}$$

### 2.2. Test Dataset and Data Curation

Two compounds were selected for model testing and validation—Oxycodone and Buprenorphine. They were selected because of their well characterized PK and existence of in vivo data for both transdermal (TD) and non-TD routes that could be utilized for optimizing parameters and coefficients critical for predicting localized distribution/penetration. Compound physicochemical properties and in vivo plasma concentration time profiles included in this study were extracted from publicly available datasets published in peer-reviewed journals. The drug parameters used for simulation and the datasets used for model training are reported in Table 1 and Table 2, respectively.

### 2.3. Subjects

Parameters characterizing subject-specific physiological behavior (e.g., tissue flow rates, tissue volume, tissue composition) for the different physiological compartments in the BIOiSIM model were adapted from reputed literature sources [33,34,35]. For the purpose of this study, and to maximize clinical relevance of simulation, only compound PK datasets from humans were included.

### 2.4. Statistical Analysis

The statistical methods utilized for assessment of model performance and optimization have been described in previous work [11]. The in vivo plasma concentration datasets were sourced from publicly available literature and manually digitized from source publications using “WebPlotDigitizer” version 4.2.34. Error bars were included where available; when unavailable, the error for each individual point was assumed to be equivalent to the reported % SD reported for C_max_. Model extension, development, and validation were done in Python with Cython integration in conjunction with auxiliary packages matplotlib (v2.0.2) and Numpy (v1.14.2). Model validation and analysis of model goodness-of-fit/accuracy was conducted using four quantitative metrics: absolute average fold error (AAFE), average fold-error (AFE), geometric mean fold-error (GMFE) across the pharmacokinetic outputs and ratio paried t-test to assess statistically-significant differences in the curve fits. The output parameters predicted specifically for this study using the BIOiSIM model include: C_max_, AUC_0–t_, AUC_0–∞_, AUMC_0–t_, MRT, and Vd_ss_ and are calculated using well-established relationships. In lieu of using the PK values reported in the original publications, each of these statistics was recalculated using noncompartmental methods from the digitized plots to minimize discrepancy. For each PK output, AAFE, AFE, and GMFE were calculated as:

(12) $$AFE=Average\;fold\;error=10^{\frac{1}{n}\overset{n}{\underset{i=1}{\sum }}\log\left(\frac{Predicted_{i}}{Observed_{i}}\right)\;}$$

(13) $$AAFE=Absolute\;average\;fold\;error=10^{\frac{1}{n}\overset{n}{\underset{i=1}{\sum }}\left|\log\left(\frac{Predicted_{i}}{Observed_{i}}\right)\right|\;}$$

(14) $$GMFE=Geometric\;mean\;fold\;error={\left(AAFE_{AUC}\times AAFE_{tmax}\times AAFE_{Cmax}\right)}^{\frac{1}{3}}$$

where *n* is the total number of compounds used in the analysis, and *Predicted*_i_/*Observed_i_* correspond to predicted and observed values of PK parameters, respectively. A statistical test comparing simulated plasma concentration profiles relative to experimental measurements was done using ratio paired t-test. Calculations of *AFE*, *AAFE*, test statistics, and visual analysis were done in GraphPad Prism version 8.4.3 (GraphPad Software, San Diego, CA, USA) and Microsoft Excel (2016).

## 3. Results

### 3.1. Simulation Accuracy

**Figure 3:**
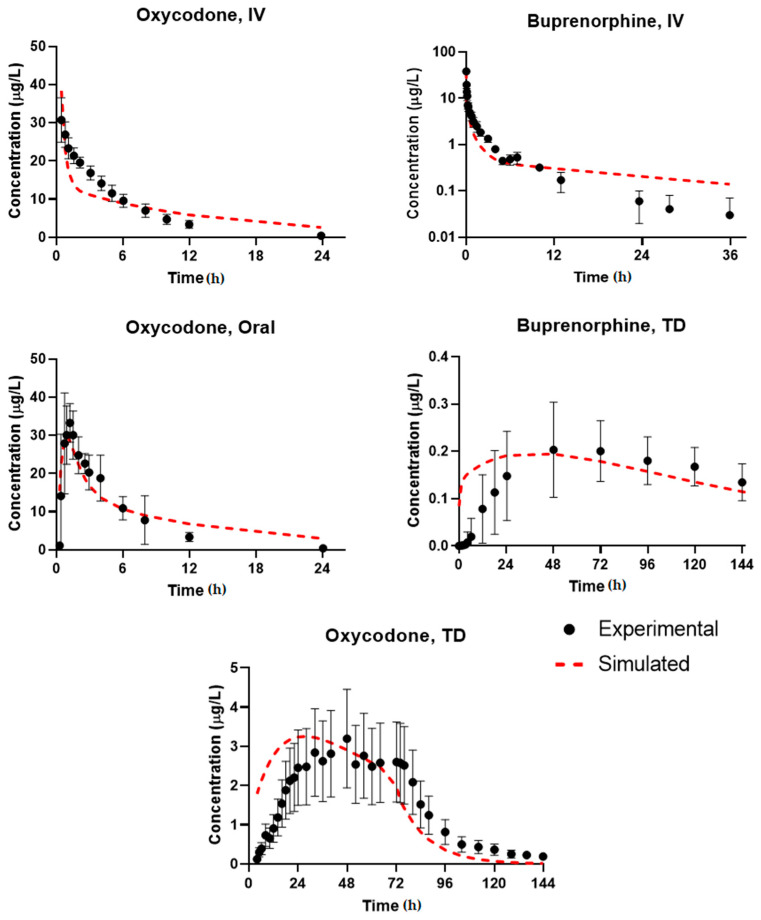
Dose and route-of-administration dependent prediction of compound plasma concentration for Oxycodone and Buprenorphine. Dashed lines correspond to BIOiSIM simulation outputs. Error bars and individual data points were digitized from the original publications, where available, and correspond to standard error/standard deviation, as presented in the original work.

The performance of the base transdermal model and systems biology PK model was evaluated by simulating the systemic plasmavenous concentration of compounds Oxycodone and Buprenorphine in healthy humans (Figure 3). For Oxycodone, three datasets were obtained from literature (IV, Oral, TD) routes whereas for Buprenorphine two robust datasets were identified (IV, TD). Additional datasets were identified for both compounds for in vivo disposition and PK property estimation; however, they were excluded during the data curation phase as a result of inconsistent data reporting and issues with units. The optimized and experimental values used for simulation of drug plasma pharmacokinetics are summarized in Table 1.

Accuracy of the simulation was evaluated by visual assessment of the plots, calculation of AAFE for three key metrics (AUC_0–t_, C_max_, t_max_), an average GMFE, and a *p*-value via t-test. Results from the analysis are summarized in Table 3. Overall, the simulation results were highly accurate for both compounds—GMFE was less than 1.3 for the three PK outputs studied, indicating an average of less than 1.3 fold-error for the fits. 

For Buprenorphine, visual analysis of the IV and TD plots indicated that the simulation results were overall accurate with some discrepancies in the TD profile. The ascending phase of Buprenorphine administration was predicted with higher variability than the terminal phase; the visible time lag in plasmavenous concentration post-administration may indicate that this is a result of under-predicting long-term skin retention of the compound. This likely leads to the lower *p*-value calculated via the paired *t*-test (0.0112, significant difference) for the regression profile. Additionally, the IV profile appears to have the opposite problem—at lower concentrations, the simulation appears to under-predict clearance. Given that these simulations are fitting a mean parameter set, this is not surprising, especially with the variable clearance profile of Buprenorphine in patients. The AAFE for AUC_0–t_, C_max_, and t_max_ were <1.2 for IV and <1.3 for TD, indicating that although some specific characteristics of the curve profiles may be underfit, the overall systemic exposure is adequately and accurately simulated. GMFE for IV and TD (1.07, 1.10 respectively) supports the overall integrity of the fit. It is expected that although there may be inter-individual variance in the transdermal permeation rate, the relative permeability of compound at each skin layer will maintain their proportionality relative to the mean optimized rate as derived in the Methods section and, thus, are still useful for simulating and estimating compound exposure at each layer.

For Oxycodone, the visual analysis of the fits indicated that the simulation was well within the error of the experimentally measured datapoints. The terminal phase for the IV plot do evidence some under-prediction of clearance (and slight over-prediction of distribution), and as with Buprenorphine this can be speculated to be a result of the mean parameter fit to the data versus a true population simulation. Overall, all ROA’s and datasets fit well, as indicated by the high *p*-values–0.75, 0.17, and 0.87 for IV, Oral, and TD, respectively. GMFE values are <1.25 for all three PK outputs (corresponding to low fold-error), with the largest AAFE in the t_max_ for the transdermal route. Given the high experimental variability observed in the datapoints, it is likely that the t_max_ shown in the averaged plot from the original source may be skewed by an outlier; thus, the fit can overall be considered high-accuracy and utilized for interpolating the skin concentration achieved during administration and to simulate changes in permeability as a function of skin lesion presence.

### 3.2. Predicted Skin Model Parameters

As expected, the more lipophilic buprenorphine had a higher optimized permeability than oxycodone (5.93 × 10^−4^ vs. 1.00 × 10^−4^, respectively). The partition coefficients and effective permeabilities of the individual skin layers—stratum corneum, viable epidermis, dermis—are shown in Table 4. These values were predicted from an input of the optimized *k_perm_* value (“Total”), and using models developed by Kretsos et al. [24] for dermis/viable epidermis diffusivity and Potts, Guy for stratum corneum diffusivity [22]. Using Equation (2) and the estimated thicknesses for each layer, permeabilities were calculated from these simulated conditions, added, and re-normalized to maintain the proportionality between the calculated stratum corneum and dermis permeability.

For both compounds, stratum corneum (SC) resistance to permeation was significant. In the case of the highly lipophilic buprenorphine (logP 4.98), the stratum corneum was less of a significant barrier, and the thickness of the skin layer was a bigger driving of resistance (~60% of the resistance came from the dermis). For oxycodone, the low lipophilicity (logP = 0.255) resulted in a calculated resistance for the stratum corneum that was significantly higher than the other layers (99.5%). 

### 3.3. Simulation of Disposition in Patients with Lesions

**Figure 4:**
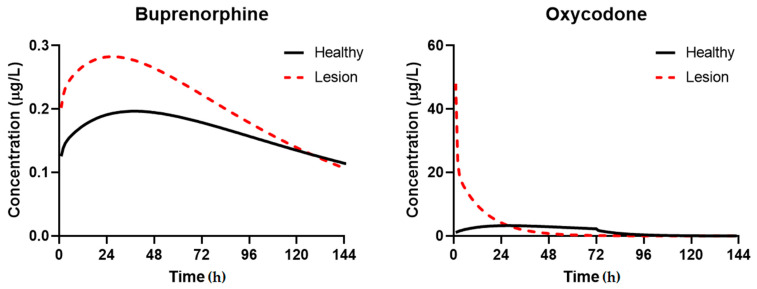
Prediction of the systemic disposition of Buprenorphine and Oxycodone in patients with healthy and compromised skin (lesions). Dosing regimens used for simulation were the same as cited in Table 4.

The simulations of plasmavenous concentration using the dosage regimens documented in Table 2 in patients with lesions relative to healthy skin are shown in Figure 4. For Buprenorphine, the increase in C_max_ and AUC_0–t_ were modest (~50% change in C_max_, ~30% in AUC_0–t_), with an overall decline over seven days more rapidly than in healthy patients as a result of the faster permeation and effective clearance. For Oxycodone, the C_max_ was significantly higher with rapid elimination as a function of the rapid permeation.

**Figure 5:**
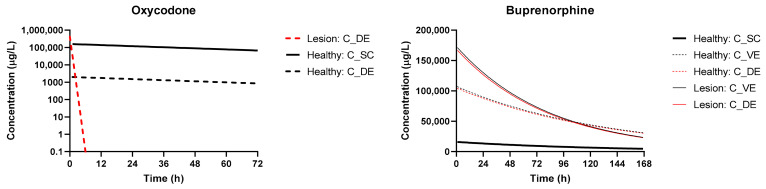
Prediction of skin layer concentrations for compounds administered in healthy patients and patients with lesions. The Oxycodone dataset excludes Viable Epidermis (VE) concentration plots, as the high permeability of that layer results in a concentration profile that is virtually identical to the concentration at apical dermis surface.

Figure 5 captures the effects of skin lesions and skin layer depth on the concentration of compounds. For buprenorphine, the majority of compound permeates rapidly through the stratum corneum and partitions into the dermis, where the concentration slowly declines as the reservoir in the compound vehicle is depleted. Thus, the impact of lesions is not as significant (<2-fold change in C_max_, AUC_0–t_). This contrasts to the hydrophilic oxycodone, where the rate-limiting stratum corneum layer prevents a significant portion of the compound from permeating deeper in healthy patients. At the surface of the dermis, used as a relative site-of-action for opioid receptor agonism, the concentration of oxycodone sharply declines over the first 6 h of exposure with a significantly higher peak.

## 4. Discussion

In recent years, the topical use of analgesics of various drug classes has gained popularity as a painless and noninvasive route of delivery with simple and easy administration [2,36] Clinical studies have demonstrated the analgesic efficacy of opioids when applied topically in certain situations such as skin ulcers and oral mucositis [1]. The advantage of administering an opioid topically is the avoidance of systemic adverse effects such as nausea, constipation, and sedation. Generally, opioid analgesics, when applied on skin, show the properties of transdermal drugs that are absorbed into the blood flow and exert systemic influence, in particular, activation of the opioid receptors in the central nervous system. There are several practices, formulations, and dose concentrations of the topical opioid preparations proposed for clinical use [37]. There are three drug compounds among the central opioid analgesics that exert topical influence and possess low skin permeability, i.e., Morphine, Diamorphine, and Buprenorphine. Despite growing evidence since the 1990s that they are efficient and safe in acute and chronic pain conditions, only Buprenorphine was shown to be free of serious adverse effects [38].

Buprenorphine is an agonist of mu-opioid receptors showing no marked interaction with both delta- and kappa-receptors. Clinical trials indicate greater pain relief, improvement of sleep quality, and decreased need for rescue therapy, when Buprenorphine is used for cancer pain treatment, which confirm the promising safety profile of this drug [36]. It is of special interest because of the long period of action and high lipophilic properties, suggesting beneficial characteristics for the topical use [39]. Demonstration of peripheral opioid receptors in inflamed synovia supports the concept of the local mechanisms of analgesia for this drug. A Buprenorphine patch was shown to provide relief in the treatment of moderate to severe cancer pain as well as musculoskeletal pain syndrome [17]. Short-term analgesic effect due to topical administration of 100 μg Buprenorphine after knee arthroscopy was shown to be non-inferior to 0.25% bupivacaine [17]. Our results confirm sufficient accumulation of Buprenorphine in epidermis and dermis layers of the skin where major part of the peripheral opioid receptors is located. The results given in the Table 4 suggest that Buprenorphine meets much less resistance being transferred through SC in comparison to Oxycodone. On the other hand, it has higher capacity of retention in the viable epidermis and dermis because it shows high permeability and lower resistance coefficients. These parameters suggest lower systemic exposure of Buprenorphine in comparison to Oxycodone, which is proved by the plasma concentration versus time plots provided in Figure 5. 

Oxycodone was also used as a topical analgesic and demonstrates sufficient clinical efficacy despite it is not clear if those effects were central or caused by stimulation of the skin opioid receptors [18]. In the present study, we have demonstrated that Oxycodone has good retention in healthy skin, thus, providing analgesics activity. On the other hand, in a case of skin lesion, predictions have shown dramatic decrease of the Oxycodone concentration in the skin layers suggesting rapid transdermal absorption and low topical analgesic activity. 

Acute skin lesion modeling was performed by removal of the SC parameters from the prediction computation because generally SC is partly or completely disrupted in patients with acute lesions [40]. This approach is confirmed by the experimental and clinical studies showing desquamation or total disintegration of SC in acute thermal injury [41], partial disruption in the patients with pressure ulcers II-III degree [40], and even in atopic dermatitis involuntary scratching provoked by severe itching can lead to a physical disruption of the SC, thereby exacerbating the intrinsic weakness in the barrier [27,42].

Several key insights can be drawn from the simulated results and profiles. The first is that indeed, as expected, the presence of topical lesions has an impact on both the systemic and local disposition of compounds. This effect appears to be more pronounced in low-lipophilicity compounds that typically permeate less effectively across an intact stratum corneum. This is an indication that Oxycodone in a fixed-release formulation (e.g., a patch, not a lotion) may be preferred in patients with topical lesions in the context of pharmacokinetics. The formulation matrix can act as a release rate limiting factor taking over the main function of stratum corneum. In addition, transdermal dosage forms with controlled drug release mechanisms can be tuned meeting the desired drug systemic exposure level without compromising skin absorption. Such further work to investigate these phenomena by expanding the analysis to more structurally-diverse compounds, as well as conducting in vitro verification of compound partitioning and penetration to specific sites using Franz diffusion cells or in vivo via tape-stripping methods, could contribute the deeper insight [14]. Extending the model to a full brick-and-mortar simulation of the stratum corneum to isolate potential influence of transcellular diffusion and retention, as well as permeation through the sebum could provide interesting insight when coupled with further experimental datasets.

Additional assumptions in the model may require further experimentation to characterize variability in simulations between subjects and compounds. In the model, intra-skin metabolic/clearance effects are assumed to be dominant in viable epidermis and dermis layers and are, thus, captured in the bioavailability values used in the model for each compound. Further in vitro experiments may also be relevant to evaluating the impact of these mechanisms on transdermal compound retention and local exposure. Given the impact of skin hydration levels on compound ionization in skin, sensitivity analyses that explore changes in transdermal permeability among multiple patients can give useful insight into the impact these properties have on compound permeation. 

There are some results published that indicate epicutanious administration of opioid analgesics, in particular morphine, is followed by rapid drug absorption into the systemic circulation to the levels that are comparable with plasma concentration profile noted injection of oral intake [43]. The present study is focused on clarifying the possible benefits associated with the topical use of opioid analgesics applied either on healthy skin or on impaired skin with disrupted superficial layers. Topical use does not suppose systemic exposure of the buprenorphine and oxycodone leading to substantially lower risk of adverse reaction with relatively high analgesic activity. In particular, they do not provoke development of the immune system impairment making them promising candidates for the analgesic therapy. 

The results of the present study have shown Buprenorphine to have much higher skin retention capacity in comparison to Oxycodone, which can be explained by its greater lipophilicity [44]. That difference in the absorption rate was more obvious in the model of the damaged skin, as it supposes the lack SC barrier function accelerating drug permeability. Values of the skin resistance indicate that the main barrier for Oxycodone is SC layer. Therefore, disruptions of this layer result in almost completely reduced resistance and dramatically accelerated absorption rate with the following rapid systemic exposure. These findings suggest that among these two test drugs, Buprenorphine should be considered a drug of choice for topical administration in the treatment of acute pain syndrome in damaged skin. Selection and characterization of the opioid analgesic drug with a topical type of activity is considered to be quite promising, as it would allow for effective pain management with the low risk of adverse reactions. 

## 5. Conclusions

In the present study, the BIOiSIM platform was used to demonstrate the potential benefits and drawbacks of topical opioid agonist use such as Buprenorphine and Oxycodone for management of topically presented severe pain syndrome. The results have confirmed that both these drugs have good skin retention and can stimulate opioid receptors located in the lower layers of epidermis, mostly in stratum basale. Oxycodone has poorer accumulation capacity in the skin in the presence of lesions, whereas Buprenorphine shows good topical properties in any skin conditions. These results suggest that opioid analgesics with substantial skin retention capacity can be applied topically for the treatment of pain syndrome in a case of skin impairment associated with severe pain. Drugs with poor retention and, thus, a great level of transdermal absorption, such as Oxycodone, are recommended to be avoided in the topical treatment of pain in acute skin lesions. Evaluation of the computational results and experimental data indicate that computational tools, such as BIOiSIM, have utility in conjunction with experimental data for assessing and optimizing dosing strategies.

## Figures and Tables

**Table 1:** Drug properties and PK parameter values used in simulations of Oxycodone and Buprenorphine disposition. Values were obtained from literature, default value approximation, or machine leaning (ML) optimization. Values marked with (*) were optimized.

Property	Oxycodone	Buprenorphine
Chemical class	Weak base	Weak base
Behavior	Full opioid agonist	Opioid agonist-antagonist
Plasma protein fraction unbound (fu,p)	0.55	0.04 [29]
Blood:plasma binding coefficient (B:P)	1.0465 *	0.55 [30]
Clearance [L/h]	28.3 *	85.5 *
Tissue:plasma partition coefficient (Kp)	5.29 *	13.3464
Transdermal permeability (kperm) [cm^2^/h]	0.0001 *	0.000593 *
Bioavailability (Oral)	0.735	N/A
Bioavailability (TD)	0.3312 *	0.15 [31]
First-order absorption rate constant (ka)	0.8281 *	N/A
logP	0.255 [32]	4.98 [32]
pKa acid	13.56	7.5
pKa base	8.21	12.54

**Table 2:** Background on datasets used for systemic plasma-venous compartment disposition simulation and optimization of missing PK parameters.

Drug Name	ROA	Dosage	Experimental Setup
Oxycodone	IV	0.1 mg/kg	12 healthy subjects were given 0.1 mg/kg of intravenous oxycodone after pre-treatments with placebo.
	Oral	15 mg	15 mg oral dose of the drug was administered to 12 healthy volunteers.
	Transdermal	23.4 mg, Patch	Subjects received a single 3-day (72 h) application of three 40 cm^2^ solid matrix oxycodone transdermal patches containing 6.7 mg tocopheryl phosphate mixture and 23.4 mg of oxycodone per patch.
Buprenorphine	IV	1.2 mg	Buprenophine in a single dose 1.2 mg was administered to six healthy male volunteers.
	Transdermal	1.68 mg	A buprenorphine transdermal system delivering 10 mcg/hour was applied to healthy volunteers.

**Table 3:** Comparison of model predicted outputs to experimental outputs.

	Compounds	Buprenorphine		Oxycodone		
Output Metric	ROA	IV	TD	IV	Oral	TD
	Dose (mg)	1.2	1.68	6.75	39	23.4
AUC_(0–t)_ (µg*h/L)	Observed *	NA	NA	NA	180.00	209.05
	Calculated	17.45	25.99	153.10	180.29	214.01
	Predicted	14.83	26.27	161.28	208.86	220.04
	AAFE	1.18	1.01	1.05	1.16	1.03
	AFE	1.18	0.99	0.95	0.86	0.97
C_max_ (µg/L)	Observed *	37.52	NA	NA	38.00	3.40
	Calculated	38.16	0.20	30.73	33.26	3.20
	Predicted	36.76	0.20	38.41	29.95	3.26
	AAFE	1.04	1.03	1.25	1.11	1.02
	AFE	1.04	1.03	0.80	1.11	0.98
T_max_ (h)	Observed *	0.04	NA	NA	1.08	49.30
	Calculated	0.04	48.02	0.43	1.19	47.99
	Predicted	0.04	37.99	0.43	0.86	27.25
	AAFE	1.00	1.26	1.00	1.39	1.76
	AFE	1.00	1.26	1.00	1.39	1.76
Statistics	GMFE	1.07	1.10	1.10	1.21	1.23
	*p*-value	0.4474	0.0112	0.7468	0.1666	0.8696
	Significant difference? (*p*-value < 0.05)	No	Yes	No	No	No

* Outputs marked as “observed” are those that were pulled directly from the source validation data manuscripts. “Calculated” corresponds to recalculation of output values using internal non-compartmental methods. Predicted C_max_ values correspond to the maximum sampling concentration within the time range of observed timepoints.

**Table 4:** Calculated parameters for skin disposition model.

Parameter Calculated		Oxycodone	Buprenorphine
Partition Coefficients (K)	SC/w	3.73	5.29
	De/w	10.29	57.29
	VE/SC	2.76	10.83
Permeability (*k_perm_*) [cm/h]	Total	1.000 × 10^−4^	5.93 × 10^−4^
	SC	1.005 × 10^−4^	15.6 × 10^−4^
	VE	0.82	0.04
	Dermis	220 × 10^−4^	9.88 × 10^−4^
Resistance (R) [h/cm]	Total	10,000	1686.82
	SC	9952.99	641.39
	VE	1.21	26.98
	Dermis	45.49	1011.71

## Data Availability

Data is contained within the article.

## Supplementary Materials

The following are available online at https://www.mdpi.com/1999-4923/13/2/284/s1, Table S1: Main assumption of the skin physiological characteristics used for prediction of opioid skin absorption.
