# Supplementary material for: In Silico Simulation of the Systemic Drug Exposure Following the Topical Application of Opioid Analgesics in Patients with Cutaneous Lesions
Source: Pharmaceutics. 2021 Feb 21;13(2):284. doi: 10.3390/pharmaceutics13020284 (PMC7924840; doi:10.3390/pharmaceutics13020284)
Supplement: Supplementary file 1 [file pharmaceutics-13-00284-s001.pdf]

pharmaceutics

MDPI

# Supplementary Materials:

# In Silico Simulation of the Systemic Drug Exposure Following the Topical Application of Opioid Analgesics in Patients with Cutaneous Lesions

**Maksim Khotimchenko, Victor Antontsev, Kaushik Chakravarty, Hypatia Hou and Jyotika Varshney**

**Table S1.** Main assumption of the skin physiological characteristics used for prediction of opioid skin absorption.

| Assumption | Source |
|---|---|
| One-dimensional Fickian diffusion is the dominant transport mechanism for the compounds selected. | [1] |
| Stratum corneum diffusion is dominated by lipid channels, and is a static barrier at a constant hydration level. | [2] |
| The formulation is assumed to be well-mixed. | [2] |
| Viable epidermis:dermis partition is equal to 1. | [3] |
| Vehicle:skin partitioning is equivalent to water:skin. | [4,5] |
| The plasma compartment is an infinite sink in the diffusion model of transdermal compound permeation. | [4] |
| In patients with lesions, stratum corneum is disrupted. The viable epidermis and dermis remain intact. | [6] |
| Stratum corneum does not significantly impact compound clearance or compound retention. | [7] |
| The compounds are not significantly cleared or retained within the skin. | [8] |
| The cornified layer (stratum corneum) of the skin is the main barrier to compound diffusion for intact skin. | [4] |

## References


1. Goyal, N.; Thatai, P.; Sapra, B. Surging footprints of mathematical modeling for prediction of transdermal permeability. *Asian J Pharm Sci* **2017**, *12*, 299-325, doi:10.1016/j.ajps.2017.01.005.
2. Lian, G.; Chen, L.; Han, L. An evaluation of mathematical models for predicting skin permeability. *J Pharm Sci* **2008**, *97*, 584-598, doi:10.1002/jps.21074.
3. Polak, S.; Ghobadi, C.; Mishra, H.; Ahamadi, M.; Patel, N.; Jamei, M.; Rostami-Hodjegan, A. Prediction of concentration-time profile and its inter-individual variability following the dermal drug absorption. *J Pharm Sci* **2012**, *101*, 2584-2595, doi:10.1002/jps.23155.
4. Kretsos, K.; Miller, M.A.; Zamora-Estrada, G.; Kasting, G.B. Partitioning, diffusivity and clearance of skin permeants in mammalian dermis. *Int J Pharm* **2008**, *346*, 64-79, doi:10.1016/j.ijpharm.2007.06.020.
5. Mitragotri, S.; Anissimov, Y.G.; Bunge, A.L.; Frasch, H.F.; Guy, R.H.; Hadgraft, J.; Kasting, G.B.; Lane, M.E.; Roberts, M.S. Mathematical models of skin permeability: an overview. *Int J Pharm* **2011**, *418*, 115-129, doi:10.1016/j.ijpharm.2011.02.023.
6. Harding, C.R. The stratum corneum: structure and function in health and disease. *Dermatol Ther* **2004**, *17 Suppl 1*, 6-15, doi:10.1111/j.1396-0296.2004.04s1001.x.
7. Nicoli, S.; Bunge, A.L.; Delgado-Charro, M.B.; Guy, R.H. Dermatopharmacokinetics: factors influencing drug clearance from the stratum corneum. *Pharm Res* **2009**, *26*, 865-871, doi:10.1007/s11095-008-9785-y.
8. Heilmann, S.; Kuchler, S.; Schafer-Korting, M. Morphine metabolism in human skin microsomes. *Skin Pharmacol Physiol* **2012**, *25*, 319-322, doi:10.1159/000342067.
